# Telemedicine in geriatric oncology is here to stay

**DOI:** 10.3389/fmed.2024.1439975

**Published:** 2024-10-01

**Authors:** Koshy Alexander, Amy L. Tin, Sincere McMillan, Farnia Amirnia, Heidi Yulico, SungWu Sun, Beatriz Korc Grodzicki

**Affiliations:** ^1^Memorial Sloan Kettering Cancer Center, New York, NY, United States; ^2^Weill Cornell Medicine, Cornell University, New York, NY, United States

**Keywords:** geriatric oncology, telemedicine, interdisciplinary, risk assessment, telehealth, geriatric assessment

## Abstract

**Introduction:**

Advancing age is the most important risk factor for cancer. Collaborations with medical and surgical-oncology divisions, and supportive services are required to assist older adults with cancer through their assessment and treatment trajectories. This often requires numerous clinical encounters which can increase treatment burden on the patient and caregivers. One solution that may lighten this load is the use of telemedicine.

**Methods:**

At Memorial Sloan Kettering, the Cancer and Aging Interdisciplinary Team (CAIT) clinic risk stratifies and optimizes older adults planned for medical cancer treatment. We analyzed patients seen in the CAIT clinic between May 2021 and December 2023, focusing on their utilization of telemedicine, and on the differences in characteristics of the visits and the results of the Geriatric Assessment based on visit type.

**Results:**

Of the 288 patients (age range 67–100) evaluated, the majority (77%) chose telemedicine visits. Older age, lower educational status, living in New York City, abnormal cognitive screen, impaired performance measures, IADL dependency and having poor social support were all associated with choosing an in-person visit as opposed to telemedicine.

**Conclusion:**

Older patients with cancer frequently choose and can complete telemedicine visits. Efforts should be directed to develop an infrastructure for remote engagement, improving reach into rural and underserved areas, decreasing the burden generated by multiple appointments.

## Introduction

The world’s population is aging rapidly. The number of Americans ages 65 and older is projected to increase from 58 million in 2022 to 82 million by 2050 (47% increase) and the 65-and-older age group’s share of the total United States (US) population is projected to rise from 17 to 23% (1). Advancing age is the most important risk factor for cancer. The incidence rates for cancer overall climb steadily as age increases, from fewer than 25 cases per 100,000 people in the age group under 20, to more than 1,000 per 100,000 people in age groups 60 years and older (2).

There are six commonly accepted dimensions of treatment burden based on a conceptual framework including financial, medication, administrative, time/travel, lifestyle, and healthcare (3). While the solutions to some of these problems may require population based and governmental support strategies, some can be tackled on individual basis. A study looking at caregiver burden in caregivers of older adults with cancer identified two significant factors-employment status of the caregiver and the caregiver’s perspective of the patient’s functional dependency (4).

Management of cancer in older adults is different than that in younger age groups. The heterogeneity in the aging process along with the accumulation of medical comorbidities and their consequences makes this a complex process. Collaborations with multiple medical and surgical-oncology divisions, and supportive services are required to assist older adults with cancer through their assessment and treatment trajectories. This often requires numerous clinical encounters and tests. If all these encounters are in-person, they can increase treatment burden on the patient (time/travel, financial difficulties, lifestyle). The numerous visits potentially increase caregiver burden as well, if the patient requires assistance to get to these, especially if the caregiver must take time off work to accompany the patient. One solution that may lighten this load is the use of telemedicine.

The COVID-19 pandemic ushered in rapid adoption of telemedicine. Data from the COVID-19 supplemental survey of the National Health and Aging Trend Study in 2020 showed that telehealth use in adults over 65 years of age increased from 4.6% pre-pandemic to 21.1% (5). Older adults’ knowledge and familiarity with technology, access to devices, web connectivity and setting up for visits were all sited as concerns related to telehealth. A nation-wide survey of clinicians in the US (7,246 responses) was conducted in March 2022. They felt that older adults may opt out of use of telehealth services; some of the reasons were: the technology may not align with older adults’ preferences and abilities (49%), older adults’ physical and cognitive challenges (48%), older adults’ preference to be seen in person (47%), lack of access to technology and/or connectivity (45%), family/caregivers’ preferences against the use telehealth (42%), and older adults’ privacy concerns (40%) (6). Nevertheless, the perceived advantages of telehealth in the care of older adults, may outweigh the challenges, highlighting an opportunity in clinical care.

Therefore, we asked whether the older patients with cancer referred to our Cancer and Aging Interdisciplinary Team (CAIT) clinic (7), when presented with both options (in-person vs. remote visit), would be prone to choose one over the other.

## Materials and methods

Memorial Sloan Kettering Cancer Center (MSK) is an academic cancer treatment and research institution. It is a large tertiary care center and provides care to patients from all over the US as well as international patients. The main campus is in Manhattan, New York City (NYC), but its 24-site network spans large areas across the states of New York and New Jersey.

**Figure 1:**
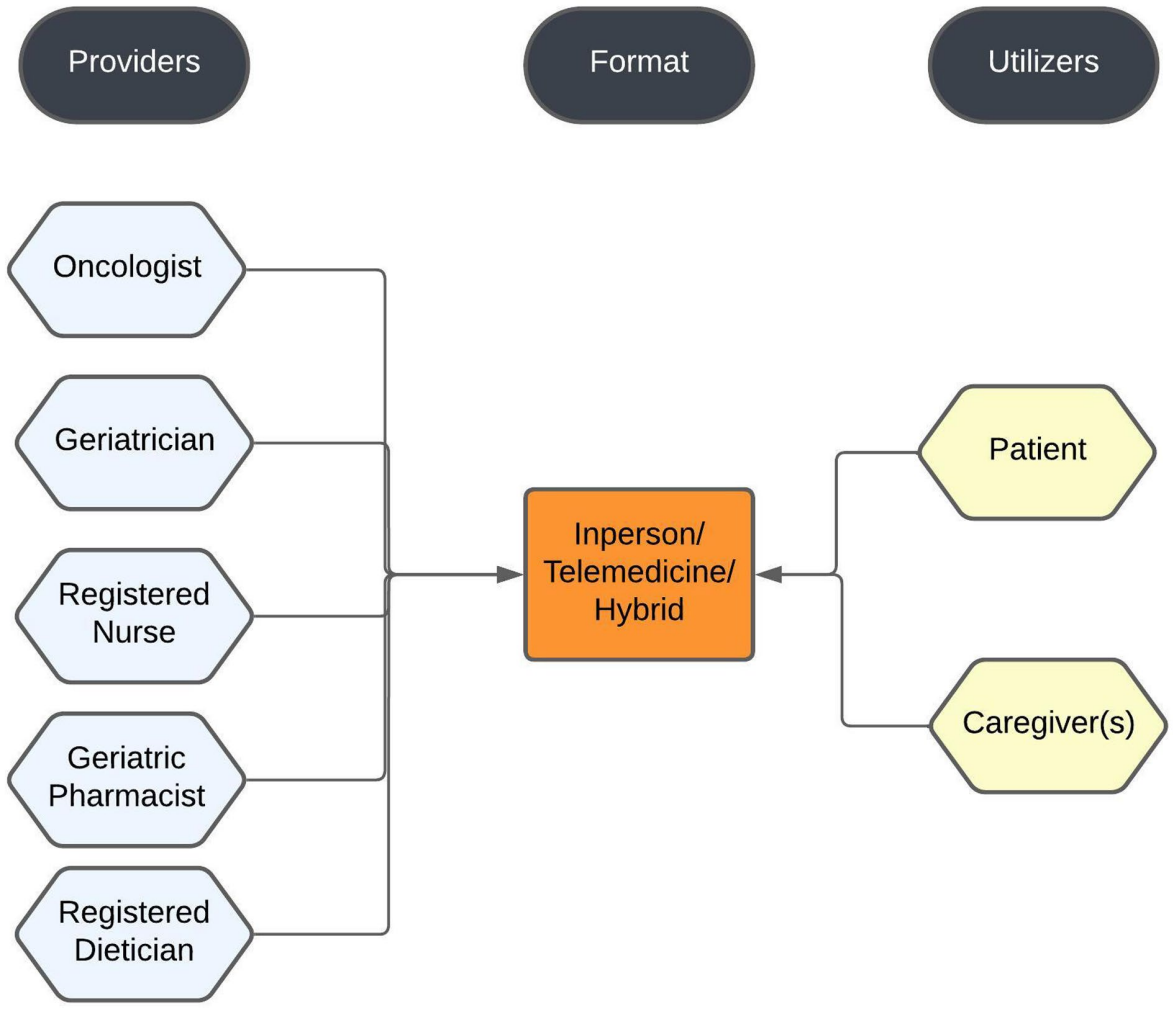
CAIT clinic components.

We have previously published on the development and implementation of the CAIT clinic model (7) for older patients with cancer being planned for medical cancer treatment and referred by their oncologists for risk assessment, and in this current study aim to further describe the patients seen in this clinic. Referral criteria to the CAIT clinic were age ≥ 65, with any of the following: oncologists’ impression of frailty, multimorbidity and/or cognitive impairment. The criteria were intended to be more inclusive as previous studies have already demonstrated benefits of Geriatric Assessment in patients planned for cancer treatment. The components of the CAIT clinic are shown in Figure 1. The model allows patients to choose the service format; in-person, telemedicine (remote) or a combination (hybrid). The current platform we use for telemedicine is a *Microsoft™ Teams application* that can be accessed by the patients through the MSK patient portal. Patients who opt for telemedicine may be in their home (“direct-to-patient” style) or go to the nearest MSK regional center to be set up for a “hub-and-spoke” style telemedicine visit. In the hub and spoke format, patients traveled to a regional MSK location from where they used telemedicine to meet with CAIT clinic clinicians located in Manhattan. Some patients manage the process on their own and some involve a caregiver or family member. Family members/health care agents have the option to join the visit remotely from a location different from the patient’s location. Once the appointment is scheduled the patient receives an online questionnaire through the patient portal called the electronic Rapid Fitness Assessment (eRFA), a patient-reported, electronic version of the Geriatric Assessment developed and used by the MSK Geriatrics Service since 2015 (8).

The hybrid, flexible clinic structure allows asynchronous assessments to be conducted by the interdisciplinary team of clinicians on the same or consecutive days. Each team member completes a discipline-specific assessment (Table 1), the results of which are compiled by the Geriatrician and discussed with the Oncologist.

**Table 1:** CAIT clinical assessments.

Clinician	Domain/disease assessed	Tools
Geriatric pharmacist	Medication review	Medication Appropriateness Index (MAI) (33) Lexicomp (34) Beer’s Criteria (35)
Registered dietitian	Nutrition	Mini Nutritional Assessment (MNA) (36)***
Registered nurse	Patient reported Geriatric Assessment items	Electronic Rapid Fitness Assessment (eRFA) (8)
Clinical psychologist	Distress Mood, support	
Geriatrician	Function	Activities of daily living (ADLs) (37) Instrumental activities of daily living (IADLs) (38)
	Cognition*	Montreal Cognitive Assessment (MoCA) (39) Mini-Cog (40)
	Performance Measures**	Timed-Up and Go Test (TUG) (41) 30 s chair stand (42)
	Medical Comorbidities	
Oncologist	Cancer diagnosis and treatment	

^*^Among patients who had both cognitive tests, we utilized the results of the MoCA (abnormal defined as < 26/30) over the Mini-Cog (abnormal < 3/5).

^**^For patients who had both performance measures, results of the 30 s chair stand test (abnormal based on age/gender cutoffs) was selected for the telemedicine cohort, and the TUG (abnormal > 10 s) for in-person cohort.

^***^For the MNA, a score < 12/14 was considered abnormal.

In this retrospective study of a prospectively maintained dataset, we describe all consecutive patients seen in the CAIT clinic between May 2021 and December 2023. We first focused on the utilization of telemedicine. We then reported characteristics related to the visit and impairments captured on the eRFA, based on whether patients utilized a telemedicine format. We present frequency (proportion) for categorical variables and median (interquartile range) for continuous variables. For select characteristics of interest and domains of the eRFA, we used a two-sample test for equality of proportions and provided the corresponding 95% confidence interval (CI) around the difference to test for differences between the patients whose chose an in-person CAIT clinic visit vs. patients who chose a telemedicine CAIT clinic visit. Missing data were assumed not to be ‘missing not at random’ and therefore whether details were available considered independent of how patients would respond. Moreover, all patients seen in the CAIT clinic undergo detailed evaluation of each Geriatric Assessment domain, regardless of service format, therefore we do not anticipate any missing characteristics to be related to this choice.

The eRFA had to be completed within 3 months prior to and up to 1 week after the CAIT clinic visit date (relevant eRFA). Although some patients had completed an eRFA outside this time frame, for a different clinic visit, those responses were not included in our analysis since they would not accurately reflect the patient’s status during the CAIT clinic visit.

All analyses were conducted using R version 4.3.2 with the tidyverse (v2.0.0) and gtsummary (v1.7.2) packages (9–11).

## Results

Between May 2021 and December 2023, a total of 288 consecutive patients were evaluated in the CAIT clinic. The median number of days from referral to visit was 12 (IQR 8, 18) days. Patient and clinical characteristics are shown in Tables 2, 3, stratified by visit type. Overall, the median age of patients at the time of their visit was 80 (IQR 76, 85; range 67, 100). Among our cohort, 66 (23%) appointments occurred in-person, 215 (75%) visits were through the direct-to-patient telemedicine format, and 7 (2.4%) were in the hub and spoke telemedicine format. Therefore, a total of 222 patients had a telemedicine visit.

**Table 2:** Patient characteristics.

	In Person, *N* = 66	Telemedicine, *N* = 222
Geriatric Visit Format		
Direct to patient telemedicine	0 (0%)	215 (97%)
Hub and spoke telemedicine	0 (0%)	7 (3.2%)
In Person	66 (100%)	0 (0%)
Age at Visit		
65–69	2 (3.0%)	3 (1.4%)
70–74	5 (7.6%)	50 (23%)
75–79	18 (27%)	68 (31%)
80–84	26 (39%)	44 (20%)
85–89	12 (18%)	42 (19%)
90–100	3 (4.5%)	15 (6.8%)
Sex		
Female	47 (71%)	150 (68%)
Male	19 (29%)	72 (32%)
Race		
White	43 (72%)	151 (87%)
Black	11 (18%)	11 (6.4%)
Asian	1 (1.7%)	9 (5.2%)
Other	5 (8.3%)	2 (1.2%)
Unknown	6	49
Education status		
Less than high school diploma	11 (18%)	8 (4.3%)
High school diploma	13 (21%)	36 (20%)
Some college	7 (11%)	31 (17%)
College graduate	15 (24%)	49 (27%)
Advanced degree	16 (26%)	60 (33%)
Unknown	4	38
Relationship status		
Partnered	24 (39%)	112 (61%)
Not Partnered	38 (61%)	73 (39%)
Unknown	4	37
Living Situation		
Living Alone	21 (32%)	57 (26%)
Living with 24/7 Aide or Skilled Nursing Facility	1 (1.5%)	6 (2.7%)
Living with Family	44 (67%)	157 (71%)
Unknown	0	2
Primary Language		
English	45 (73%)	180 (97%)
Not English	17 (27%)	5 (2.7%)
Unknown	4	37
Geographical Residence		
New York City	43 (65%)	40 (18%)
New York State (Outside of New York City)	12 (18%)	104 (47%)
New Jersey	6 (9.1%)	57 (26%)
Connecticut	0 (0%)	9 (4.1%)
Other state in USA	4 (6.1%)	12 (5.4%)
Outside USA	1 (1.5%)	0 (0%)

Data are presented as *N* (%).

**Table 3:** Clinical characteristics.

	In Person, *N* = 66	Telemedicine, *N* = 222
Type of Cancer		
Breast	13 (20%)	33 (15%)
Gastrointestinal	9 (14%)	46 (21%)
Genitourinary	9 (14%)	13 (5.9%)
Gynecological	8 (12%)	73 (33%)
Head and Neck	1 (1.5%)	4 (1.8%)
Hematological	9 (14%)	18 (8.1%)
Hepato-pancreato-biliary	12 (18%)	31 (14%)
Melanoma	1 (1.5%)	0 (0%)
Other	4 (6.1%)	4 (1.8%)
Cognitive testing		
Abnormal result	36 (61%)	77 (36%)
Normal result	23 (39%)	134 (64%)
Unknown	7	11
Performance measures		
Abnormal result	45 (74%)	83 (42%)
Normal result	16 (26%)	116 (58%)
Unknown	5	23
Nutritional status		
Abnormal result	36 (55%)	95 (43%)
Normal result	30 (45%)	125 (57%)
Unknown	0	2
Polypharmacy (≥10 Medications)		
Yes	26 (39%)	106 (48%)
No	40 (61%)	115 (52%)
Unknown	0	1

Data are presented as *N* (%).

Ninety-one percent (60/66) of in-person visits had a relevant eRFA, while 74% (165/222) of telemedicine visits had a relevant eRFA, corresponding to an overall completion rate of 78%. Among patients who completed the eRFA, the median time from survey start to submission was 12 (IQR 8, 24) minutes, and over half of patients (58%) completed the survey by themselves, a fifth of patients completed it with assistance from someone else, and the remaining (22%) surveys were completed by someone else entirely.

Table 4 presents select patient characteristics based on whether patients had an in-person or telemedicine visit, with percentages reported being among patients with the same visit type. In contrast, Supplementary Table 1 presents the percentages among patients with the same characteristic of interest. For example, among patients who were 80 years or older, the majority proceeded to telemedicine (71% vs. 29%; Supplementary Table 1), however when considering the breakdown of age among each visit type, a larger proportion of patients who came for an in-person visit were 80-years or older compared to patients who had a telemedicine visit (62% vs. 45%; difference = 17%; 95% CI 2.2, 31%; *p* = 0.026; Table 4). Among the patients with an abnormal cognition test, under a third (32%) came for an in-person visit, and the remaining (68%) visited through a Telemedicine platform. Unsurprisingly, a larger proportion of patients who live in the NYC area came in-person as opposed to telemedicine (52% vs. 48%), though the comparison between groups is more meaningful when considering that 65% of in-person visits vs. 18% of telemedicine visits were patients who resided in NYC (difference = 47%; 95% CI 34, 61%; *p* < 0.001).

**Table 4:** Comparison of select characteristics based on visit format.

	*N*	In person, *N* = 66	Telemedicine, *N* = 222	Difference	95% CI	*p*-value
Age ≥ 80 at visit	288	41 (62%)	101 (45%)	17%	2.2, 31%	0.026
Female	288	47 (71%)	150 (68%)	3.6%	−9.9, 17%	0.7
Living alone	286	21 (32%)	57 (26%)	5.9%	−7.7, 20%	0.4
High school diploma or less	246	24 (39%)	44 (24%)	15%	0.12, 29%	0.037
NYC residence	288	43 (65%)	40 (18%)	47%	34, 61%	<0.001
Abnormal result from cognition test	270	36 (61%)	77 (36%)	25%	9.4, 40%	0.001
Abnormal result from performance measures	260	45 (74%)	83 (42%)	32%	18, 46%	<0.001
Abnormal nutritional status	286	36 (55%)	95 (43%)	11%	−3.3, 26%	0.14
Polypharmacy (≥10 Medications)	287	26 (39%)	106 (48%)	−8.6%	−23, 5.9%	0.3

Patients in the telemedicine group includes “Direct to patient telemedicine” and “Hub and spoke telemedicine.” Differences presented are proportion of telemedicine visits from proportion of in-person visits. Data are presented as *N* (%).

Table 5 compares the prevalence of impairments on the eRFA based on whether patients had an in-person visit compared to a telemedicine visit. We saw evidence that a larger proportion of patients who had their visit in-person had poor social support (63% vs. 44% of telemedicine patients; difference 19%; 95% CI 3.6, 35%; *p* = 0.017) and IADL dependency (75% vs. 57%; difference 18%; 95% CI 3.6, 32%; *p* = 0.021), though results should be interpreted in the context of multiple testing.

**Table 5:** Prevalence of impairments on the eRFA domains based on visit type among patients who completed a relevant eRFA.

	*N*	In person, *N* = 60	Telemedicine, *N* = 165	Difference	95% CI	*p*-value
ADL dependency	225	42 (70%)	103 (62%)	7.6%	−7.3, 22%	0.4
IADL dependency	225	45 (75%)	94 (57%)	18%	3.6, 32%	0.021
KPS ≤80	225	34 (57%)	76 (46%)	11%	−5.2, 26%	0.2
History of fall	225	34 (57%)	76 (46%)	11%	−5.2, 26%	0.2
Poor social support	225	38 (63%)	73 (44%)	19%	3.6, 35%	0.017
Limited social activity	224	45 (75%)	129 (79%)	−3.7%	−17, 10%	0.7
Major distress	202	29 (54%)	71 (48%)	5.7%	−11, 23%	0.6
Depression	225	41 (68%)	107 (65%)	3.5%	−11, 18%	0.7
Weight Loss >10 lbs	209	21 (38%)	39 (25%)	12%	−3.6, 28%	0.13

Patients in the Telemedicine group includes “Direct to patient telemedicine” and “Hub and spoke telemedicine.” Differences presented are proportion of telemedicine visits from proportion of in-person visits. Data are presented as *N* (%).

## Discussion

This study assessed whether older patients that were referred for geriatric evaluation prior to starting on systemic cancer treatment chose in-person visits or telemedicine visits, and the association between patients’ characteristics and this choice. Our study looking at encounters between mid-2021 and end of 2023, therefore occurred mostly in the post-pandemic period when patients were offered the choice. Our results show that most patients (77%; 95% CI 72, 82%) chose the telemedicine option and were able to complete it with some help when needed. Older age, lower educational status, living in NYC, abnormal cognitive screen, impaired performance measures, IADL dependency and having poor social support were all associated with a higher likelihood of choosing an in person visit as opposed to telemedicine.

Historically the healthcare sector has limited patients’ choice on the format in which they utilize the service. Even with patient-centered-care (12), we require patients to physically come to the clinical setting where we provide the specific assessments and interventions. In this sense, patient-centered-care is more about patient’s choice in healthcare utilization and shared decision making rather than a choice about how they access the healthcare services (13). Some comprehensive care models require that patients choose providers from within the program (e.g., Program of All-Inclusive Care for the Elderly (PACE) (14, 15)) limiting the reach of their healthcare coverage, in order to receive the benefits of the program. The complex nature of healthcare systems worldwide and the wide variety of programs available for different needs with their own specific regulations, make healthcare navigation very complex for patients. Not all patients are able to comprehend the navigation requirements, leading to disorientation, futile and stressful searches, uncertainty, and discontinuities in health care (16).

COVID-19 opened the healthcare industry to the adoption of telemedicine out of necessity. During the pandemic both patients and providers were forced onto platforms that neither group was comfortable with. Additionally, these early telemedicine platforms were rather rudimentary. This was not purely the fault of the technological companies. Prior to the pandemic, the healthcare industry had high entry barriers (17) that disincentivized startup companies from developing products and services in this area. Only well-funded established corporations were prepared to enter this highly regulated world. Even the few products and services that were available rarely ventured into areas that included protected health information (PHI). Most dealt with Domotics (18) and Robotic technology (19).

Only after the pandemic restrictions eased, did utilizing telemedicine really became a choice to patients. Pre-pandemic, this was not offered for the most part, and during the pandemic telemedicine was the only way to see providers. It is important to keep in mind that choosing telemedicine did not place any restrictions on the reach of their insurance coverage or on the variety of providers they could see, i.e., it did not require additional healthcare system knowledge and navigation skills to sign up for.

A commissioned survey examining behaviors of US adults over 70 conducted by Independa, a platform for remote engagement, found that older adults are increasingly embracing telehealth, with a notable surge in adoption rate to 97% in 2023 up from 86% in 2022 and 75% in 2021 (20). The rate from our results falls in line with these estimates, with our cohort of patient ranging from 67 to 100 years of age. In a recent study looking at patients older than 18, age 75 and older was negatively associated with telemedicine use (21). Similarly in our study we found that age 80 and over was positively associated with choosing in-person visits.

In our study, among patients who chose to come in, the rate of having poor social support was significantly higher than among patients who chose telemedicine (difference 19%; 95% CI 3.6, 35%; *p* = 0.017). The patients’ discomfort with technology or the unease in tackling a new process, coupled with a lack of support would prevent these individuals to get set up for telemedicine. The share of people living alone increased every decade from 1940 to 2020. In 2020, 11% of US households were one-person households 65 years and older. Nearly 16 million people aged 65 and older in the US lived solo in 2022, three times as many who lived alone in that age group in the 1960s. Older adults living alone are more prevalent in rural counties (22). Our study did not find evidence of a difference in the choice of visit format in patients living alone vs. living with family. This could mean that even if patients live alone, they have either the capacity themselves or adequate support to set up a telemedicine visit.

Not surprisingly, we found a significant difference in patients living in NYC choosing in-person visits. The geographic proximity to the clinical locations very likely played a role in this choice. This is general consumer behavior (23). Studies have already identified having a high school diploma or less as a significant barrier to telehealth use (24). Our findings align with this.

Patients with instrumental activities of daily living (IADL) dependency were expected to favor the use of telemedicine potentially lightening caregiver burden. However, our data shows that patients with IADL impairment are significantly more likely to choose an in-person visit. These patients may already have an established system in place for in-person clinic visits compensating for their functional dependency (25). Rather than try out a new method and develop a new process, they may have by default relied on resources they have in place already such as the New York State Paratransit ridership program which returned to normal much faster than fixed route ridership after the pandemic (26). This trend could change soon if these patients become more familiar with the use of telemedicine platforms; or in the near future when relatively fitter and younger patients already familiar with the technology and process, develop infirmities. The significance of impaired performance tests and cognitive assessments, also follows the same line of thought: patients with gait impairment and cognitive impairment likely have a support system in place to access healthcare the traditional way (27).

Clinicians have been using telehealth in the care of older adults, across clinical roles, sites, and purposes suggesting that perceived advantages of telehealth outweigh challenges in the care of older adults (6). However, there appears to be a potential disconnect between clinicians and their older adult patients regarding the level of concern for the use of telehealth. Although nearly half (49%) of older adults surveyed in the Healthy Aging poll noted privacy concerns as a reason to avoid telehealth pre-pandemic, this proportion fell to 24% in June 2020 (28). However, 40% clinicians surveyed felt that privacy would be a major barrier and reason their older patients would not participate in telehealth (6). These contrasting views suggest that the perspective of older patients has shifted since the pandemic and clinicians should be aware of this shift.

Our study is not without limitations. This is a select population of patients predominantly white, mostly well-educated and living in the US northeast. Higher educational status (24) and being white (29) are known factors associated with higher telemedicine usage and therefore likely contributes to the result that over two-thirds of our patients opted for a telemedicine visit. The US Northeast enjoys the best overall internet speeds of any region in the country and the slowest speeds in the Northeast are still above the Federal Communications Commission’s (FCC) minimum threshold (30). Hence the results may not align with results from institutions in other geographic areas with different patient populations, as due to subpar internet speeds elsewhere, patients may opt for an in-person visit over telemedicine. This is a retrospective study and only 78% of the cohort completed the eRFA. As with all retrospective studies, our results are dependent on the availability and accuracy of the data. However, our dataset is prospectively maintained, undergoing chart review to ensure the accuracy of the data. The availability of an easy-to use telemedicine platform cannot be generalized. Since the pandemic, MSK has built its own telemedicine platform which is more user friendly than some of the commercially available software. MSK has good institutional tech support, and the office and clinic staff walk patients through the process of connecting to a visit when needed. This kind of support may not be available at all institutions.

As with any newly developing product or service, the technology behind telehealth will continue to improve based on user experience data, making telemedicine a smoother operation. Perhaps we are seeing this process already evolving from the rudimentary platforms and early prototypes used during the pandemic to the current ones in existence. Older adults are now more familiar with telemedicine platforms and visit formats, and practice obtained from repeat visits will make it easier for most of them. Hence, it’s important for clinicians to keep in mind that patient perspectives on telemedicine will also continue to change as the world of telemedicine evolves. From a public health policy perspective Medicare coverage of telehealth is a potential issue. It was initially granted as a temporary emergency measure due to the need for social distancing during the COVID-19 pandemic. In December 2022, Congress extended Medicare coverage of telehealth visits through the end of 2024 (31, 32). On the US clinician survey, two thirds of the clinicians said that they would eliminate or reduce telehealth services if the Medicare reimbursement waivers expired (6). A new equilibrium remains to be established from a policy perspective as well as from the perspective of technology growth, user experience, patient/caregiver preference, and health care utilization.

## Conclusion

In this cohort of 288 patients aged 65 and older with cancer, who had the choice of an in-person visit vs. a telemedicine visit, the ample majority chose telemedicine, especially those who lived far from the institution. Efforts should be directed to develop an infrastructure for remote engagement, improving reach into rural and/or underserved areas and decreasing the burden generated by multiple appointments.

## Data Availability

The original contributions presented in the study are included in the article/Supplementary material, further inquiries can be directed to the corresponding author.

## References

[1] Mather MSP. Fact sheet: aging in the United States. Washington DC: Population Reference Bureau (2024).

[2] Age and Cancer risk: National Cancer Institute; (2021) Available at: https://www.cancer.gov/about-cancer/causes-prevention. Accessed May 28, 2024

[3] Sav A McMillan SS Akosile A. Burden of treatment among elderly patients with Cancer: a scoping review. Healthcare. (2021) 9:612. doi: 10.3390/healthcare9050612 34069688 PMC8160635

[4] Hsu T Loscalzo M Ramani R Forman S Popplewell L Clark K. Factors associated with high burden in caregivers of older adults with cancer. Cancer. (2014) 120:2927–35. doi: 10.1002/cncr.28765, PMID: 24898093 PMC4159406

[5] Choi NG DiNitto DM Marti CN Choi BY. Telehealth use among older adults during COVID-19: associations with sociodemographic and health characteristics, technology device ownership, and technology learning. J Appl Gerontol. (2022) 41:600–9. doi: 10.1177/07334648211047347, PMID: 34608821 PMC8847316

[6] Wardlow L Roberts C Archbald-Pannone L. Perceptions and uses of telehealth in the Care of Older Adults. Telemed J E Health. (2023) 29:1143–51. doi: 10.1089/tmj.2022.0378, PMID: 36493377 PMC10440646

[7] Alexander K Hamlin PA Tew WP Trevino K Tin AL Shahrokni A. Development and implementation of an interdisciplinary telemedicine clinic for older patients with cancer-preliminary data. J Am Geriatr Soc. (2023) 71:1638–49. doi: 10.1111/jgs.18267, PMID: 36744590 PMC10175129

[8] Shahrokni A Tin A Downey RJ Strong V Mahmoudzadeh S Boparai MK. Electronic rapid fitness assessment: a novel tool for preoperative evaluation of the geriatric oncology patient. J Natl Compr Cancer Netw. (2017) 15:172–9. doi: 10.6004/jnccn.2017.0018, PMID: 28188187 PMC5507335

[9] Team RC. R: A language and environment for statistical computing. Vienna, Austria: R Foundation for Statistical Computing (2021).

[10] Wickham H Averick M Bryan J Chang W McGowan LD’A François R. Welcome to the tidyverse. J. Open Source Software. (2019) 4:1686. doi: 10.21105/joss.01686

[11] Daniel D Sjoberg KW Michael Curry S Lavery JA Larmarange J. Reproducible summary tables with the gtsummary package. The R J. (2021) 13:570–80. doi: 10.32614/RJ-2021-053

[12] What is patient-centered care? Catalyst carryover. Massachusetts Medical Society (2017); 3.

[13] Barry MJ Edgman-Levitan S. Shared decision making — the pinnacle of patient-centered care. N Engl J Med. (2012) 366:780–1. doi: 10.1056/NEJMp1109283, PMID: 22375967

[14] Arku D Felix M Warholak T Axon DR. Program of all-inclusive Care for the Elderly (PACE) versus other programs: a scoping review of health outcomes. Geriatrics. (2022) 7:31. doi: 10.3390/geriatrics7020031 35314603 PMC8938794

[15] Health NYSDo. About Managed Long Term Care New York. (2022). Available at: https://www.health.ny.gov/health_care/managed_care/mltc/aboutmltc.htm (Accessed May 28, 2024).

[16] Griese L Berens EM Nowak P Pelikan JM Schaeffer D. Challenges in navigating the health care system: development of an instrument measuring navigation health literacy. Int J Environ Res Public Health. (2020) 17:5731. doi: 10.3390/ijerph17165731, PMID: 32784395 PMC7460304

[17] Baker MC Stratmann T. Barriers to entry in the healthcare markets: winners and losers from certificate-of-need laws. Socio Econ Plan Sci. (2021) 77:101007. doi: 10.1016/j.seps.2020.101007

[18] Sánchez VG Taylor I Bing-Jonsson PC. Ethics of smart house welfare technology for older adults: a systematic literature review. Int J Technol Assess Health Care. (2017) 33:691–9. doi: 10.1017/S0266462317000964, PMID: 29151393

[19] Morgan AA Abdi J Syed MAQ Kohen GE Barlow P Vizcaychipi MP. Robots in healthcare: a scoping review. Curr Robot Rep. (2022) 3:271–80. doi: 10.1007/s43154-022-00095-4, PMID: 36311256 PMC9589563

[20] Telehealth survey: Quick, satisfying and boomer approved Independa (2023). Available at: https://independa.com/telehealth-survey-quick-satisfying-and-boomer-approved/. Accessed May 28, 2024

[21] Chang E Penfold RB Berkman ND. Patient characteristics and telemedicine use in the US, 2022. JAMA Netw Open. (2024) 7:e243354-e. doi: 10.1001/jamanetworkopen.2024.3354, PMID: 38517438 PMC12285594

[22] Lydia Anderson CW Rose M Kreider Thomas Gryn. Home alone: More than a quarter of all households have one person US Census Bureau (2023) Available at: https://www.census.gov/library/stories/2023/06/more-than-a-quarter-all-households-have-one-person.html (Accessed May 28, 2024).

[23] The Washington post. Location, location, location.’ How where you live influences how you shop online. Washington DC: Personal Finance (2014).

[24] Williams C Shang D. Telehealth usage among low-income racial and ethnic minority populations during the COVID-19 pandemic: retrospective observational study. J Med Internet Res. (2023) 25:e43604. doi: 10.2196/43604 37171848 PMC10185335

[25] Abdul Latiff AR Mohd S. Transport, mobility and the wellbeing of older adults: an exploration of private chauffeuring and companionship Services in Malaysia. Int J Environ Res Public Health. (2023) 20:2720. doi: 10.3390/ijerph20032720 36768086 PMC9915393

[26] Del Mastro Barry. MTA’s paratransit program: An overview. Office of the State Deputy Comptroller for the City of New York; Albany, New York: Office of the New York State Comptroller (2023)

[27] S R. The future of disability in America. Washington (DC): Institute of Medicine (US) Committee on Disability in America (2007). 20669428

[28] Malani PKJ Solway E Buis L Singer D Kirch M. Telehealth use among older adults before and during COVID-19. Ann Arbor, Michigan: University of Michigan, Innovation IfHPa (2020).

[29] Marcondes FO Normand S-LT Le Cook B Huskamp HA Rodriguez JA Barnett ML. Racial and ethnic differences in telemedicine use. JAMA Health Forum. (2024) 5:e240131-e. doi: 10.1001/jamahealthforum.2024.0131 38517424 PMC10960201

[30] Smith C. Allconnect regional broadband report. Northeastern ed. Fort Mill, South Carolina: Allconnect (2023).

[31] Godman Heidi, Staying healthy. Harvard medical school: Harvard Health Publishing. (2023). Available at: https://www.health.harvard.edu/staying-healthy/medicare-extends-coverage-of-telehealth-through-2024. Accessed May 28, 2024

[32] Medicare. Telehealth In: CMS, editor. Baltimore, Maryland: Centers for Medicare and Medicaid Services. (2024). Available at: https://www.medicare.gov/coverage/telehealth (Accessed May 28, 2024).

[33] Spinewine A Dumont C Mallet L Swine C. Medication appropriateness index: reliability and recommendations for future use. J Am Geriatr Soc. (2006) 54:720–2. doi: 10.1111/j.1532-5415.2006.00668_8.x, PMID: 16686895

[34] Lexicomp. Netherlands: Wolters Kluwer (2000). (Accessed May 28, 2024).

[35] By the 2019 American Geriatrics Society Beers Criteria® Update Expert Panel. American Geriatrics Society 2019 updated AGS beers criteria® for potentially inappropriate medication use in older adults. J Am Geriatr Soc. (2019) 67:674–94. doi: 10.1111/jgs.15767 30693946

[36] Guigoz Y Vellas B. The Mini nutritional assessment (MNA) for grading the nutritional state of elderly patients: presentation of the MNA, history and validation. Nestle Nutr Workshop Ser Clin Perform Programme. (1999) 1:3–11. 10.1159/000062967 11490593

[37] Katz S Ford AB Moskowitz RW Jackson BA Jaffe MW. Studies of illness in the aged. The index of ADL: a standardized measure of biological and psychosocial function. JAMA. (1963) 185:914–9. doi: 10.1001/jama.1963.03060120024016 14044222

[38] Graf C. The Lawton instrumental activities of daily living scale. Am J Nurs. (2008) 108:52–62. doi: 10.1097/01.NAJ.0000314810.46029.74 18367931

[39] Freitas S Simões MR Alves L Santana I. Montreal cognitive assessment: validation study for mild cognitive impairment and Alzheimer disease. Alzheimer Dis Assoc Disord. (2013) 27:37–43. doi: 10.1097/WAD.0b013e3182420bfe 22193353

[40] Borson S Scanlan JM Chen P Ganguli M. The Mini-cog as a screen for dementia: validation in a population-based sample. J Am Geriatr Soc. (2003) 51:1451–4. doi: 10.1046/j.1532-5415.2003.51465.x 14511167

[41] Nightingale CJ Mitchell SN Butterfield SA. Validation of the timed up and go test for assessing balance variables in adults aged 65 and older. J Aging Phys Act. (2019) 27:230–3. doi: 10.1123/japa.2018-0049, PMID: 30117359

[42] Jones CJ Rikli RE Beam WC. A 30-s chair-stand test as a measure of lower body strength in community-residing older adults. Res Q Exerc Sport. (1999) 70:113–9. doi: 10.1080/02701367.1999.10608028, PMID: 10380242

