# Supplementary material for: Telemedicine in geriatric oncology is here to stay
Source: Front Med (Lausanne). 2024 Oct 1;11:1439975. doi: 10.3389/fmed.2024.1439975 (PMC11473358; doi:10.3389/fmed.2024.1439975)
Supplement: Supplementary file 1 [file Table_1.DOCX]

**Supplementary Table 1.** Comparison of select characteristics based on visit format. Patients in the Telemedicine group includes “Direct to patient telemedicine” and “Hub and spoke telemedicine”. Data are presented as N (row %).

|  | **In Person**, N = 66 | **Telemedicine**, N = 222 |
| --- | --- | --- |
| Age at Visit |  |  |
| < 80 | 25 (17%) | 121 (83%) |
| ≥ 80 | 41 (29%) | 101 (71%) |
| Sex |  |  |
| Male | 19 (21%) | 72 (79%) |
| Female | 47 (24%) | 150 (76%) |
| Living Situation |  |  |
| Not Living Alone | 45 (22%) | 163 (78%) |
| Living Alone | 21 (27%) | 57 (73%) |
| Education |  |  |
| High School Diploma or Less | 24 (35%) | 44 (65%) |
| Higher than High School Diploma | 38 (21%) | 140 (79%) |
| Geographical Residence |  |  |
| New York City | 43 (52%) | 40 (48%) |
| Outside New York City | 23 (11%) | 182 (89%) |
| Cognition test |  |  |
| Normal | 23 (15%) | 134 (85%) |
| Abnormal | 36 (32%) | 77 (68%) |
| Performance measures |  |  |
| Normal | 16 (12%) | 116 (88%) |
| Abnormal | 45 (35%) | 83 (65%) |
| Nutritional status |  |  |
| Normal | 30 (19%) | 125 (81%) |
| Abnormal | 36 (27%) | 95 (73%) |
| Polypharmacy (≥10 Medications) |  |  |
| No | 40 (26%) | 115 (74%) |
| Yes | 26 (20%) | 106 (80%) |
